# A 0.026 mm^2^ Time Domain CMOS Temperature Sensor with Simple Current Source

**DOI:** 10.3390/mi11100899

**Published:** 2020-09-28

**Authors:** Sangwoo Park, Sangjin Byun

**Affiliations:** Division of Electronics and Electrical Engineering, Dongguk University, Seoul 04620, Korea; psw7478@naver.com

**Keywords:** temperature sensor, time domain, threshold voltage, poly resistor, temperature error, CMOS integrated circuits

## Abstract

This paper presents a time domain CMOS temperature sensor with a simple current source. This sensor chip only occupies a small active die area of 0.026 mm^2^ because it adopts a simple current source consisting of an *n*-type poly resistor and a PMOS transistor and a simple current controlled oscillator consisting of three current starved inverter delay cells. Although this current source is based on a simple architecture, it has better temperature linearity than the conventional approach that generates a temperature-dependent current through a poly resistor using a feedback loop. This temperature sensor is designed in a 0.18 μm 1P6M CMOS process. In the post-layout simulations, the temperature error was measured within a range from −1.0 to +0.7 °C over the temperature range of 0 to 100 °C after two point calibration was carried out at 20 and 80 °C, respectively. The temperature resolution was set as 0.32 °C and the temperature to digital conversion rate was 50 kHz. The energy efficiency is 1.4 nJ/sample and the supply voltage sensitivity is 0.077 °C/mV at 27 °C while the supply voltage varies from 1.65 to 1.95 V.

## 1. Introduction

Due to their small form factor, low power consumption, low cost and convenient digital interface capability, integrated temperature sensor chips have been widely used for thermal monitoring in various smart applications such as the smart farm, smart factory, smart health, and so on. Integrated temperature sensor chips can be implemented in various ways depending on the sensing element which is generally chosen from the threshold voltage, mobility, resistance or thermal voltage; the measured signal type, which is generally chosen from the voltage, current, frequency, or delay time; and the overall architecture which is basically determined by the chosen sensing element and the measured signal type [1,2,3,4,5,6,7,8,9,10,11,12,13,14,15,16,17,18,19,20,21,22,23,24,25,26,27,28,29,30].

Among the possible architectures, a time domain architecture based on frequency or delay time is promising because it can benefit from the recent trend that time resolution is being increased while voltage resolution is being decreased as the CMOS channel length and the supply voltage are scaled down. By measuring time domain signals such as the clock frequency, clock period or delay time, an efficient estimate of the current temperature can be obtained [1,2,3,4,5,6,7,8,9,10,11,12,13,14,15,16,17,18,19,20]. The sensing element can be chosen from the mobility, threshold voltage or resistance. Depending on the choice, the temperature linearity of a time domain CMOS temperature sensor is fundamentally limited by the temperature linearity of the chosen sensing element. In this paper, we present a time domain CMOS temperature sensor with the sensing element dependent on both the resistance of an *n*-type poly resistor and the threshold voltage of a PMOS transistor at the same time. By adopting this simple current source as a sensing element, we gain the ability to multiply two different types of temperature characteristics of the resistance of an *n*-type poly resistor and the threshold voltage of a PMOS transistor by each other and improve the temperature linearity of the temperature sensor.

This paper is organized as follows. In Section 2, we explain how this simple current source can have better temperature linearity than the conventional approach. Section 3 describes the architecture and schematics of the implemented time domain CMOS temperature sensor adopting the simple current source. Section 4 shows the simulation results and the performance comparison with the previous works found in literature. Finally, the conclusion is given in Section 5.

## 2. Temperature Linearity of the Sensing Element

**Figure 1:**
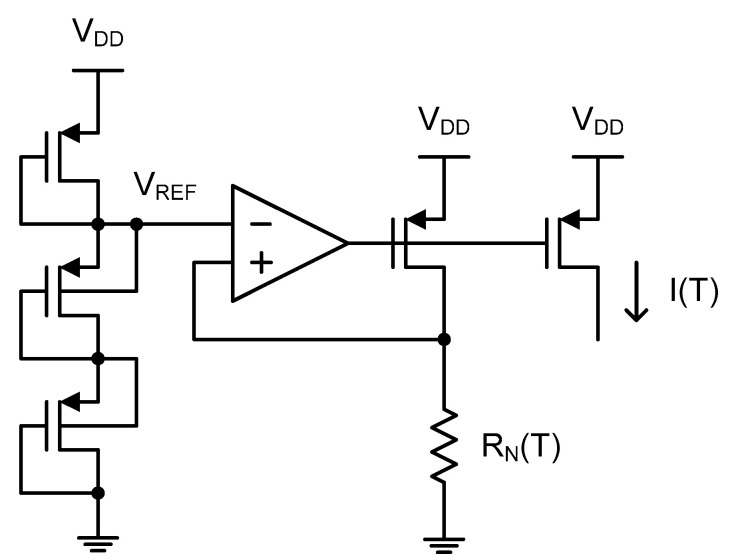
The conventional approach for generating a temperature-dependent current through a poly resistor using a feedback loop.

Figure 1 shows the conventional current source based on a single *n*-type poly resistor [1,8,9,13]. It consists of multiple diode-connected PMOS transistors between V_DD_ and the ground, a differential input to a single-ended output amplifier, an *n*-type poly resistor and a PMOS transistor. Since multiple diode-connected PMOS transistors generate a reference voltage, V_REF_, it is applied across the *n*-type poly resistor, R_N_(T), through the feedback loop consisting of the differential amplifier and the PMOS transistor as shown in the below figure. The reference current, I(T), becomes exactly proportional to V_REF_ and inversely proportional to R_N_(T) as follows. This current source has been used because its temperature linearity can be made dependent on only that of the resistor; then the analysis and design become simpler than for the other types of current sources.

(1) $$I\left(T\right)=\frac{V_{\mathrm{REF}}}{R_{N}\left(T\right)}$$

**Figure 2:**
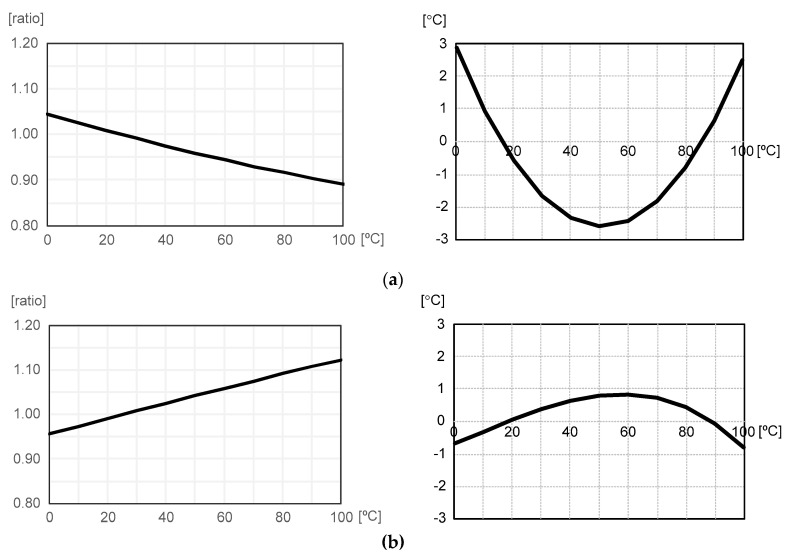
The temperature-dependent variation and temperature linearity error of (**a**) R_N_(T) and (**b**) 1/R_N_(T).

Figure 2 shows the temperature-dependent variation and temperature linearity error of R_N_(T) and 1/R_N_(T), respectively, where R_N_(T) is the resistance of the *n*-type poly resistor. In these figures, the values of R_N_(T) and 1/R_N_(T) were normalized with respect to the values measured at 25 °C and the temperature linearity error is defined as the temperature deviation, which is measured in °C, from the linear fit of R_N_(T) or 1/R_N_(T). Since R_N_(T) is convex downward whereas 1/R_N_(T) is convex upward, they can be modeled as

(2) $$R_{N}\left(T\right)=R_{N}\left(25\right)\times \left[1-a\left(T-25\right)+b{\left(T-25\right)}^{2}\right]$$

and

(3) $$\frac{1}{R_{N}\left(T\right)}=\frac{1}{R_{N}\left(25\right)}\times \left[1+\alpha \left(T-25\right)-\beta {\left(T-25\right)}^{2}\right]$$

where a, b, α and β are positive first and second order temperature coefficients of R_N_(T) and 1/R_N_(T), respectively. Here, R_N_(25) is the resistance of the *n*-type poly resistor measured at 25 °C. As shown in Figure 2, the temperature linearity error varies from −2.60 to +2.87 °C and from −0.79 to +0.83 °C for R_N_(T) and 1/R_N_(T), respectively, over the temperature range of 0 to 100 °C. This shows that 1/R_N_(T) is more linear against temperature variation than R_N_(T) if they are implemented by using this CMOS technology. Thus, in this work, we have chosen 1/R_N_(T) as the sensing element and consequently I(T) of (1) is now expressed as

(4) $$I\left(T\right)=\frac{V_{\mathrm{REF}}}{R_{N}\left(25\right)}\times \left[1+\alpha \left(T-25\right)-\beta {\left(T-25\right)}^{2}\right]$$

when the conventional current source of Figure 1 is used. Since the temperature linearity error of I(T) is limited by that of 1/R_N_(T) as implied in (4), we can expect that the temperature linearity of the temperature sensor will vary from −0.79 to +0.83 °C if there is not any other kind of additional nonlinearity source.

**Figure 3:**
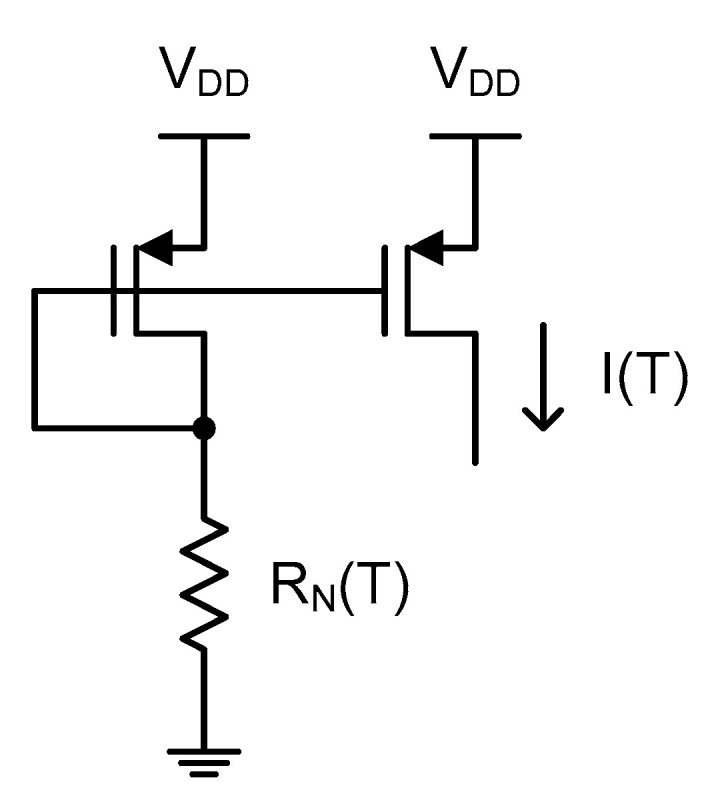
The simple current source for generating a temperature-dependent current through a poly resistor, with better temperature linearity.

Figure 3 shows the simple current source adopted alternatively in this time domain CMOS temperature sensor. As shown in the figure, it just consists of an *n*-type poly resistor, of which the resistance value is R_N_(T), and a single diode-connected PMOS transistor. Since I(T) flows through the *n*-type poly resistor and PMOS transistor, we can obtain the equation of I(T) by equating two current equations of the *n*-type poly resistor and the PMOS transistor as follows.

(5) $$I\left(T\right)=\frac{1}{2}\mu \left(T\right)C_{\mathrm{OX}}\frac{W}{L}{\left(V_{\mathrm{SG}}\left(T\right)-V_{\mathrm{TH}}\left(T\right)\right)}^{2}=\frac{V_{\mathrm{DD}}-V_{\mathrm{SG}}\left(T\right)}{R_{N}\left(T\right)}$$

Then, V_SG_(T) is solved as

(6) $$\begin{array}{cc} V_{\mathrm{SG}}\left(T\right)=V_{\mathrm{TH}}\left(T\right) & -\frac{1}{\mu \left(T\right)C_{\mathrm{OX}}\frac{W}{L}R_{N}\left(T\right)}\\ & +\sqrt{{\left(V_{\mathrm{DD}}-V_{\mathrm{TH}}\left(T\right)+\frac{1}{\mu \left(T\right)C_{\mathrm{OX}}\frac{W}{L}R_{N}\left(T\right)}\right)}^{2}-{\left(V_{\mathrm{DD}}-V_{\mathrm{TH}}\left(T\right)\right)}^{2}} \end{array}$$

Since the PMOS transistor operates in the vicinity of the threshold voltage for low power consumption in this work, V_SG_(T) can be simplified to V_TH_(T) and thus we obtain

(7) $$I\left(T\right)\approx \frac{V_{\mathrm{DD}}-V_{\mathrm{TH}}\left(T\right)}{R_{N}\left(T\right)}$$

where

(8) $$V_{\mathrm{TH}}\left(T\right)=V_{\mathrm{TH}}\left(25\right)-\gamma \left(T-25\right).$$

Here, γ is the positive temperature coefficient of the threshold voltage of the PMOS transistor and V_TH_(25) is the threshold voltage measured at 25 °C. I(T) can be finally obtained in quadratic equation form by substituting 1/R_N_(T) of (3) and V_TH_(T) of (8) into (7), as follows:

(9) $$I\left(T\right)\frac{V_{\mathrm{DD}}-V_{\mathrm{TH}}\left(T\right)}{R_{N}\left(T\right)}=\frac{V_{\mathrm{DD}}-V_{\mathrm{TH}}\left(25\right)+\gamma \left(T-25\right)}{R_{N}\left(25\right)}\times \left[1+\alpha (T-25)-{\beta (T-25)}^{2}\right]\;\;\;\;\;\;\approx \frac{V_{\mathrm{DD}}-V_{\mathrm{TH}}\left(25\right)}{R_{N}\left(25\right)}\times \left[1+A\left(T-25\right)-B{\left(T-25\right)}^{2}\right]$$

where

(10) $$A=\alpha +\frac{\gamma }{V_{\mathrm{DD}}-V_{\mathrm{TH}}\left(25\right)}$$

and

(11) $$B=\beta -\frac{\alpha \gamma }{V_{\mathrm{DD}}-V_{\mathrm{TH}}\left(25\right)}.$$

**Figure 4:**
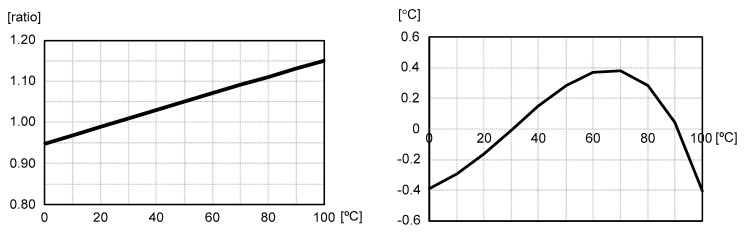
The temperature-dependent variation and temperature linearity error of I(T).

Now let us compare (9) with (4) and observe the relative increase and decrease of A and B in (10) and (11), respectively. We can see that A > α and B < β and that the temperature linearity of this simple current source has been fairly improved and the amount of the improved temperature linearity error is determined by α, β, γ and V_DD_-V_TH_(25) as indicated in (10) and (11). Contrary to the conventional approach shown in Figure 1, the reference current, I(T), of (9) depends on both the resistance of an *n*-type poly resistor and the threshold voltage of a PMOS transistor at the same time and the temperature linearity error has been successfully reduced as shown in Figure 4. The temperature linearity error of I(T) now varies from −0.40 to +0.38 °C over the temperature range of 0 to 100 °C.

## 3. Implementation

### 3.1. Architecture

**Figure 5:**
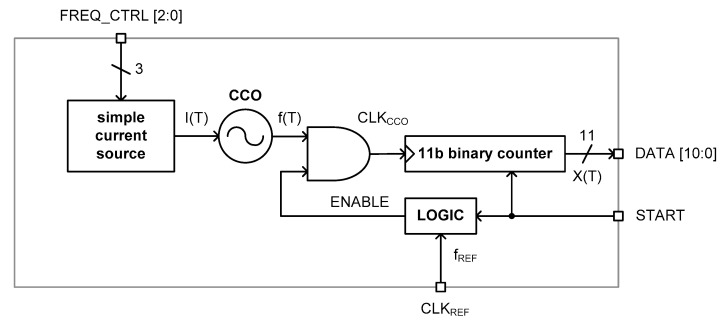
The architecture.

Figure 5 shows the architecture of the implemented time domain CMOS temperature sensor. It consists of the simple current source, a three stage current controlled oscillator (CCO), an 11b binary counter and an additional digital logic.

**Figure 6:**
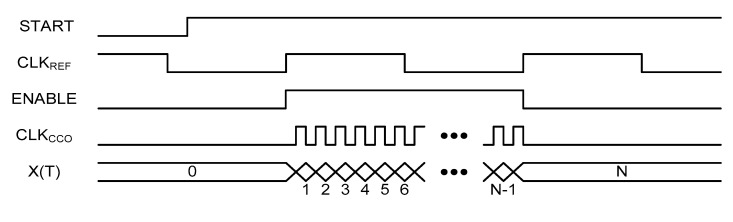
The timing diagram.

The current source generates the temperature-dependent reference current, I(T), with improved temperature linearity which is fed to the CCO. The CCO generates the clock signal oscillating at the frequency of f(T) which is linear with I(T). The logic circuit generates the ENABLE pulse whose pulse width equals one clock period, 1/f_REF_, of the external reference clock, CLK_REF_. As shown in the timing diagram of Figure 6, the rising edges of the clock signal, CLK_CCO_, generated from the CCO is counted by the 11b binary counter for the time duration of 1/f_REF_ to generate the 11b digital output code, DATA[10:0]. The DATA[10:0] is the quantized digital value of the temperature estimation function, X(T), which is defined as

(12) $$X\left(T\right)=\frac{\frac{1}{f_{\mathrm{REF}}}}{\frac{1}{f\left(T\right)}}$$

In this paper, in regard to the detailed definition of the temperature estimation function, we introduced and explained the 12 types of operational principles and the corresponding temperature estimation functions of the time domain CMOS temperature sensors from our previous work [1]. Finally, the START signal enables the 11b binary counter and the additional logic circuit to start the operation of this time domain CMOS temperature sensor.

### 3.2. Circuits

**Figure 7:**
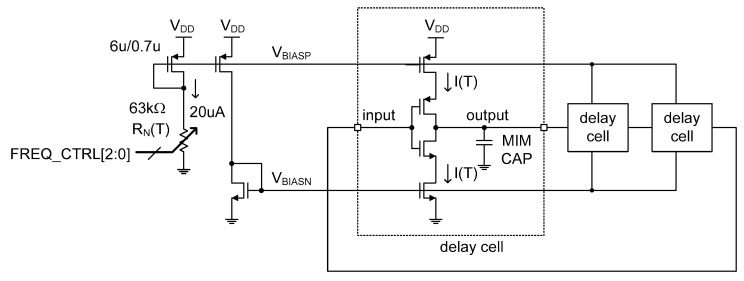
The schematic of the current source (left side) and the CCO (right side).

Figure 7 shows the schematic of the simple current source and the CCO. In the current source, the resistance of the *n*-type poly resistor, R_N_(T), has been designed to be externally tuned by the external 3b digital control word, FREQ_CTRL[2:0]. We added these extra 3b digital codes for rough compensation of the probable severe process variation of R_N_(T), which rarely happens. If the resistance of the *n*-type poly resistor, R_N_(T), is shifted too much from its typical value at the worst case corner, the total power consumption of the total temperature sensor will be affected too much. Thus, this 3b rough digital tuning by FREQ_CTRL[2:0] is only necessary for robust operation and consistent power consumption and should be carried out before we execute the two point calibration. Of course, if the resistance of the *n*-type poly resistor can be accurately controlled during fabrication within the capability of normal two point calibration, FREQ_CTRL[2:0] needs not be additionally implemented.

The temperature-dependent reference current, I(T), is delivered to each current starved inverter type delay cell inside the CCO through two DC bias voltages, V_BIASP_ and V_BIASN_, respectively. By supplying the almost equivalent currents to the PMOS and the NMOS paths of the current starved inverters, the low to high and high to low propagation delay times of each delay cell can be made almost equal to each other. Because the propagation delay time, τ(T), of each delay cell is represented as

(13) $$\tau \left(T\right)=\frac{V_{\mathrm{DD}}}{2}\times \frac{C}{I\left(T\right)}$$

the clock frequency, f(T), of the CCO can also be expressed as

(14) $$f\left(T\right)=\frac{1}{\tau \left(T\right)}\times \frac{1}{2N}=\frac{I\left(T\right)}{N\times V_{\mathrm{DD}}\times C}$$

where N is the number of total delay cells of the CCO, and f(T) is linear with I(T). Finally, the temperature estimation function, X(T), of the temperature sensor can be represented as

(15) $$X\left(T\right)=\frac{V_{\mathrm{DD}}-V_{\mathrm{TH}}\left(25\right)}{f_{\mathrm{REF}}\times N\times V_{\mathrm{DD}}\times C\times R_{N}\left(25\right)}\times \left[1+A\left(T-25\right)-B{\left(T-25\right)}^{2}\right]$$

from (9), (12) and (14).

## 4. Performance

**Figure 8:**
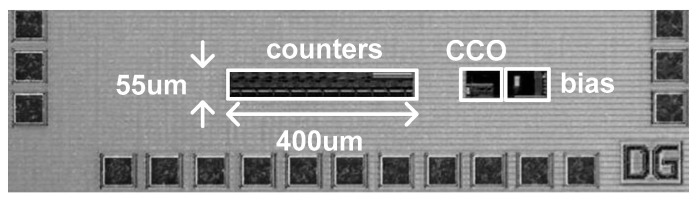
The die photo.

**Figure 9:**
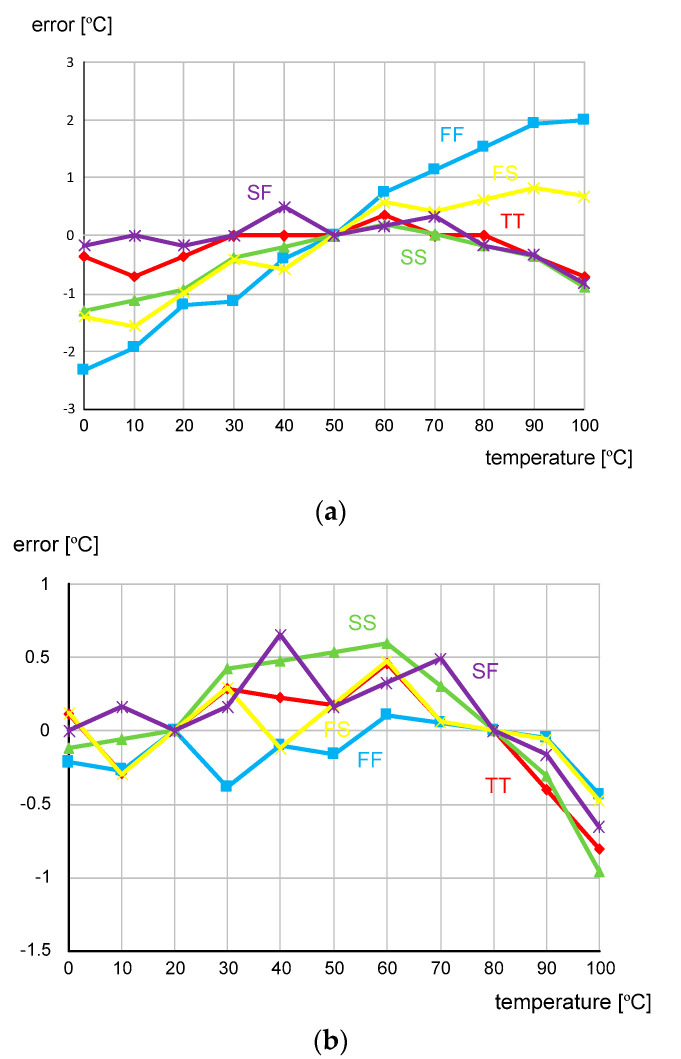
The temperature error after (**a**) one point calibration at 50 °C and (**b**) two point calibration at 20 and 80 °C, carried out against the different process corners, TT, FF, SS, FS and SF.

**Figure 10:**
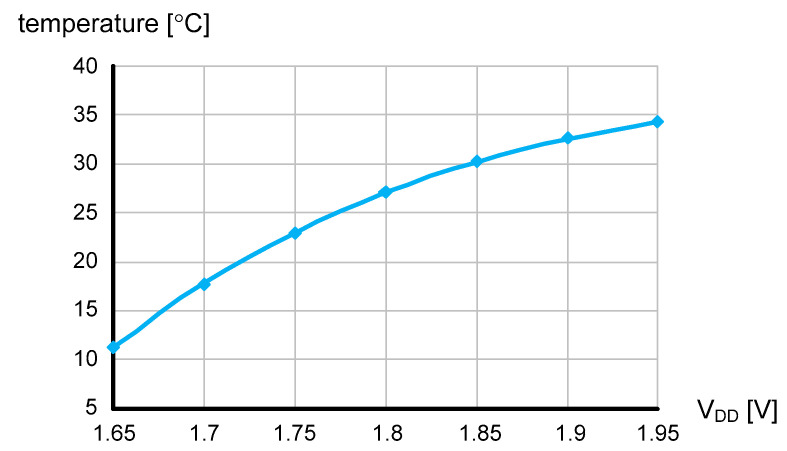
The V_DD_ sensitivity when V_DD_ is swept from 1.65 to 1.95 V.

The time domain CMOS temperature sensor was implemented in a 0.18 μm 1P6M CMOS process with a general V_TH_. Figure 8 shows the die photo and the active die area is 0.026 mm^2^. To the best of our knowledge, the die area of 0.026 mm^2^ is the smallest among the previously published time domain CMOS temperature sensors implemented in 0.18 μm CMOS technology. Figure 9 shows the simulated temperature error over the temperature range of 0 to 100 °C after one point and two point calibrations are carried out, respectively. For the process corners, TT, FF, SS, FS, and SF, the temperature error varies from −2.33 to +1.99 °C after one point calibration at 50 °C and from −0.96 to +0.66 °C after two point calibration at 20 and 80 °C, respectively. The temperature error of the time domain CMOS temperature sensor is measured as being somewhat larger than the temperature linearity error of I(T) only, because the temperature linearity error of the load capacitance, C, may also affect the temperature estimation function, X(T), as can be seen from (15). Additionally, the quantization error at the 11b digital output code, DATA[10:0], the residue process variation error after two point calibration, the approximation error of I(T) made for the derivation of (7), the charge sharing effect within the delay cell and the transistor mismatch effect between current mirrors may also be the sources of additional contributions to the increase of the total temperature error. The temperature error of Figure 9b has been measured greater than the temperature error of Figure 4. The temperature resolution was set as 0.32 °C and the temperature to digital conversion rate, which is exactly equal to f_REF_ in this design, is about 50 kHz. The energy efficiency is 1.4 nJ/sample and most of the power consumption is dissipated by the current source. As shown in Figure 10, the supply voltage sensitivity is measured as low as 0.077 °C/mV at 27 °C while V_DD_ varies from 1.65 to 1.95 V. Table 1 compares the performances of this temperature sensor with those of the previous works in literature. The previous works are all time domain CMOS temperature sensors which were implemented in CMOS processes with the same minimum channel length of 0.18 μm, for fair performance comparison with this work. Compared to the previous works, we can see that this temperature sensor occupies a relatively small die area and has relatively small temperature error as shown in Table 1.

## 5. Conclusions

We implemented a time domain CMOS temperature sensor alternatively adopting a simple current source consisting of an *n*-type poly resistor, a PMOS transistor and a simple three stage CCO. Although the current source is based on a simple architecture, it can generate a temperature-dependent reference current, I(T), which has better temperature linearity than the conventional approach because it can multiply two different temperature characteristics of the resistance of an *n*-type poly resistor and the threshold voltage of a PMOS transistor by each other. Consequently, we have successfully implemented a temperature sensor with a small die area and a small temperature error.

## Figures and Tables

**Table 1:** The performance summary.

Reference	[1]	[7]	[8]	[9]	[14]	This Work
CMOS technology	0.18 μm	0.18 μm	0.18 μm	0.18 μm	0.18 μm	0.18 μm
Die area	0.432 mm^2^	0.074 mm^2^	0.05 mm^2^	0.09 mm^2^	0.19 mm^2^	0.026 mm^2^
Supply voltage	1.8 V	0.8 V	1.0 V	1.2 V	1.2 V	1.8 V
Temperature range	0–100 °C	−20–80 °C	0–100 °C	0–100 °C	−40–85 °C	0–100 °C
Resolution	0.49 °C	0.145 °C	0.3 °C	0.3 °C	0.18 °C	0.32 °C
Accuracy	−1.6–0.6 °C	−0.9–1.2 °C	−1.6–3.0 °C	−1.4–1.5 °C	−1.0–1.0 °C	−1.0–0.7 °C
Conversion rate	25 kHz	1.2 Hz	100 Hz	33.3 Hz	1kHz	50 kHz
Energy efficiency	7.2 nJ/sample	8.9 nJ/sample	2.2 nJ/sample	2.2 nJ/sample	66.5 nJ/sample	1.4 nJ/sample
V_DD_ sensitivity	0.085 °C/mV	0.0038 °C/mV	-	0.014 °C/mV	-	0.077 °C/mV
